# Engineering *Saccharomyces cerevisiae* for Succinic Acid Production From Glycerol and Carbon Dioxide

**DOI:** 10.3389/fbioe.2020.00566

**Published:** 2020-06-26

**Authors:** Joeline Xiberras, Mathias Klein, Erik de Hulster, Robert Mans, Elke Nevoigt

**Affiliations:** ^1^Department of Life Sciences and Chemistry, Jacobs University Bremen gGmbH, Bremen, Germany; ^2^Department of Biotechnology, Delft University of Technology, Delft, Netherlands

**Keywords:** glycerol, biodiesel, NADH, succinic acid, metabolic engineering

## Abstract

Previously, our lab replaced the endogenous FAD-dependent pathway for glycerol catabolism in *S. cerevisiae* by the synthetic NAD-dependent dihydroxyacetone (DHA) pathway. The respective modifications allow the full exploitation of glycerol’s higher reducing power (compared to sugars) for the production of the platform chemical succinic acid (SA) via a reductive, carbon dioxide fixing and redox-neutral pathway in a production host robust for organic acid production. Expression cassettes for three enzymes converting oxaloacetate to SA in the cytosol (“SA module”) were integrated into the genome of *UBR2*_CBS_-DHA, an optimized CEN.PK derivative. Together with the additional expression of the heterologous dicarboxylic acid transporter DCT-02 from *Aspergillus niger*, a maximum SA titer of 10.7 g/L and a yield of 0.22 ± 0.01 g/g glycerol was achieved in shake flask (batch) cultures. Characterization of the constructed strain under controlled conditions in a bioreactor supplying additional carbon dioxide revealed that the carbon balance was closed to 96%. Interestingly, the results of the current study indicate that the artificial “SA module” and endogenous pathways contribute to the SA production in a highly synergistic manner.

## Introduction

Succinic acid (SA) has been traditionally used as surfactant, ion chelator and additive in agriculture and food (Ahn et al., 2016). Apart from these traditional applications, SA has nowadays been considered one of the most promising platform chemicals that can be produced from renewable resources (Dusselier et al., 2014; Pinazo et al., 2015). In fact, SA can be converted to a large number of chemicals including 1,4-butanediol, gamma-butyrolactone, and tetrahydrofuran as well as bio-based polymers such as polybutylene succinate (PBS) (Werpy and Petersen, 2004; Ahn et al., 2016).

Certain bacteria isolated from rumen such as *Mannheimia succiniciproducens* (Lee et al., 2002; Scholten and Dägele, 2008) and *Actinobacillus succinogenes* (Guettler et al., 1999) naturally secrete significant amounts of SA. However, these wild-type organisms are auxotrophic for several vitamins and amino acids (Beauprez et al., 2010), and the use of complex media is not feasible for economic production of bulk products. Alternatively, rational metabolic pathway engineering has been applied to well-established model bacteria such as *Escherichia coli* and *Corynebacterium glutamicum* (Jantama et al., 2008; Okino et al., 2008). Although relatively high titers and productivities have been reported, bacteria are generally considered to be sub-optimal production hosts for SA since they are susceptible to bacteriophage infections and show low tolerance towards acidity (Los, 2012). The need to maintain the pH at a neutral level requires cumbersome downstream processing and thereby generates excessive amounts of unwanted by-products as discussed by Jansen and van Gulik (2014).

Yeast and fungi are generally more tolerant to low pH and have therefore been considered as promising cell factories for the production of organic acids (Raab et al., 2010; Jansen and van Gulik, 2014; Ahn et al., 2016). The yeast species *Saccharomyces cerevisiae* and *Pichia kudriavzevii* (*Issatchenkia orientalis*) have already been successfully engineered for commercial SA production, e.g., by the companies Reverdia and Bioamber as reviewed by Ahn et al. (2016). Notably, all attempts to produce SA in *S. cerevisiae* have been based on glucose as the carbon source and SA titers and productivities are still lower than those achieved by bacterial hosts.

Three metabolic routes could be exploited for SA production in microorganisms: (i) the oxidative and (ii) the reductive branch of the tricarboxylic acid (TCA) cycle as well as (iii) the glyoxylate cycle (Raab and Lang, 2011). The reductive SA pathway has the highest possible yield since it is accompanied by the net fixation of carbon dioxide (Beauprez et al., 2010). The latter route has been established in the cytosol of a *S. cerevisiae* strain by Yan et al. (2014) who simultaneously overexpressed *PYC2* encoding pyruvate carboxylase, retargeted the peroxisomal malate dehydrogenase (*MDH3*) to the cytosol, expressed the fumarase from *E. coli* (*fumC*) and overexpressed the endogenous fumarate reductase (*FRD1*). The authors used a previously engineered baseline strain [pyruvate decarboxylase (PDC)-negative *S. cerevisiae* TAM strain] that is unable to perform alcoholic fermentation (van Maris et al., 2004). A similar cytosolic, reductive, and carbon dioxide fixing pathway has been established in *S. cerevisiae* by the company Reverdia for the commercial bio-based production of SA using glucose as the sole carbon source (van de Graaf et al., 2015, Patent No. US20150057425A1). Another study demonstrated that the additional expression of a dicarboxylic acid transporter from *Aspergillus niger* (encoded by *An*DCT-02) plays a crucial role for obtaining high SA titers and yields (Jansen et al., 2017, Patent No. US9624514B2).

**FIGURE 1:**
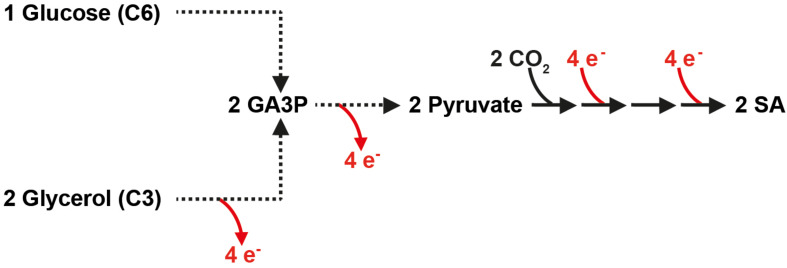
In contrast to glucose, the production of SA from glycerol via the reductive, carbon dioxide fixing SA pathway is redox-neutral. Broken lines represent several reactions. GA3P, glyceraldehyde-3-phosphate; SA, succinic acid.

It is important to note that the production of two molecules of SA from one molecule of glucose (and two molecules of carbon dioxide) via glycolysis and the reductive SA pathway is not redox-neutral and requires the input of additional electrons in the form of NADH (Figure 1). An interesting alternative carbon source for the production of SA via the attractive reductive pathway is glycerol, a molecule in which the carbon is more reduced than in glucose. As depicted in Figure 1, two molecules of SA can be produced from two molecules of glycerol (and two molecules of carbon dioxide) in a redox-neutral pathway. This allows a higher maximum theoretical yield in g SA/g substrate consumed (i.e., 1.28 g/g glycerol vs. 1.12 g/g glucose). Glycerol is a major by-product of the transesterification process during the production of biodiesel (Clomburg and Gonzalez, 2013). Although the future of the biodiesel industry is uncertain (Naylor and Higgins, 2017), the study of SA production from carbon sources that do not result in carbon catabolite repression is interesting in the view of future technologies that might implement C1 carbon sources such as methanol or formate (Cotton et al., 2020).

**FIGURE 2:**
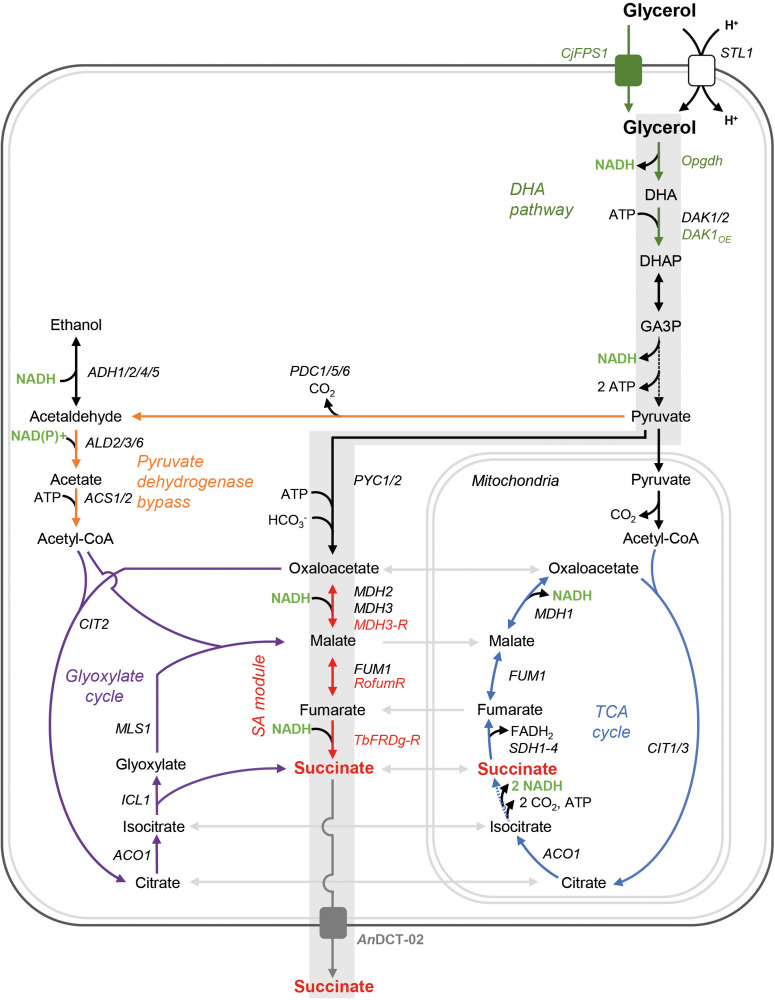
Genetic modifications for the establishment of a reductive, carbon dioxide fixing, and redox-neutral pathway from extracellular glycerol to extracellular SA in *S. cerevisiae*. Gray shading indicates the targeted pathway; however, other endogenous metabolic routes potentially leading to SA are also shown. Broken lines represent several reactions. The peroxisome is not shown. DHA, dihydroxyacetone; DHAP, dihydroxyacetone phosphate; GA3P, glyceraldehyde-3-phosphate; *STL1*, glycerol/H^+^ symporter; *DAK1/2*, dihydroxyacetone kinase; *PYC1*/*2*, pyruvate carboxylase; *MDH1*/*MDH2*/*MDH3*, malate dehydrogenase; *FUM1*, fumarase; *SDH1-4*, succinate dehydrogenase; *CIT1/2/3*, citrate synthase; *ACO1*, aconitase; *ICL1*, isocitrate lyase; *MLS1*, malate synthase; *ACS1/2*, acetyl-coA synthetase; *PDC1/5/6*, pyruvate decarboxylase; *ADH1/2/4/5*, alcohol dehydrogenase; *ALD2/3/6*, aldehyde dehydrogenase; *CjFPS1*, glycerol facilitator from *C. jadinii*; *Opgdh*, glycerol dehydrogenase from *O. parapolymorpha*; *MDH3-R*, peroxisomal malate dehydrogenase targeted to the cytosol; *RofumR*, *fumarase* from *R. oryzae*; *TbFRDg-R*, glycosomal fumarate reductase from *T. brucei* retargeted to the cytosol; *An*DCT-02, dicarboxylic acid transporter from *A. niger*.

It is important to note that the higher reducing power of glycerol can only fully be exploited for fermentative pathways in *S. cerevisiae* if the electrons are saved in the form of cytosolic NADH. However, the natural L-glycerol 3-phosphate (L-G3P) pathway transfers electrons via FADH_2_ to the respiratory chain. Our group has previously replaced the native L-G3P pathway by an alternative pathway in which dihydroxyacetone (DHA) is an intermediate (Klein et al., 2016). The respective strains utilize glycerol in an NAD-dependent manner as depicted in Figure 2.

The goal of the current study was to establish the cytosolic reductive SA pathway and the *An*DCT-02 transporter in a previously generated “DHA pathway strain” of *S. cerevisiae* and analyze the performance of the resulting strain for SA production from glycerol and carbon dioxide. A major focus of this study was to scrutinize the role of the glyoxylate cycle for SA production from glycerol in the engineered *S. cerevisiae* strain equipped with the reductive SA pathway. In fact, the key enzymes of the glyoxylate cycle have been previously demonstrated to be highly upregulated at the transcriptional level on glycerol compared to glucose (Roberts and Hudson, 2006).

## Materials and Methods

### Strains and Maintenance

The strains used in this study are listed in Table 1. Yeast cells were routinely grown on solid YPD medium containing 10 g/L yeast extract, 20 g/L peptone, 20 g/L glucose, and 15 g/L agar. Agar plates were cultivated in a static incubator at 30°C. Media were supplemented with phleomycin (20 mg/L), hygromycin B (300 mg/L), or nourseothricin (100 mg/L) for selection purposes when needed. *E. coli* DH5α was used for plasmid construction and isolation, and cells were routinely grown in lysogeny broth (LB) containing 10 g/L NaCl, 5 g/L yeast extract, 10 g/L peptone and adjusted to a pH of 7.5 with 2 M NaOH (Bertani, 1951). For selection and maintenance of plasmid containing cells, 100 mg/L ampicillin was added.

**TABLE 1:** *S. cerevisiae* strains used in this study.

Strain/modules	Genotype and genome modifications	Source or references
CEN.PK113-1A	*MAT*a	Mating type switched as described by Ho et al. (2017)
*UBR2*_CBS_-DHA	*MAT*α; *ubr2:UBR2_CBS 6412–13A_*; *gut1*:*P*_TEF1_-*Opgdh*-*T*_CYC1_-*P*_ACT1_-*DAK1*-*T*_TPS1_-*P*_PGK1_-*CjFPS1*-*T*_RPL15A_-*P*_TDH3_-*DAK1*-*T*_IDP1_	Klein et al., 2016
SA	*MATa*; YGLCτ3:*P*_PGK1_-*MDH*3-R-*T*_IDP1__–_*P_TEF1_-RofumR-T_RPL15A_*-*P*_TDH__3_*-TbFRDg-R-T_CYC1_*	This study
*UBR2*_CBS_-DHA-SA	*MAT*α; *ubr2:UBR2_CBS 6412–13A_*; *gut1*:*P*_TEF1_-*Opgdh*-*T*_CYC1_-*P*_ACT1_-*DAK1*-*T*_TPS1_-*P*_PGK1_-*CjFPS1*-*T*_RPL15A_-*P*_TDH3_-*DAK1*-*T*_IDP1_; YGLCτ3:*P*_PGK1_-*MDH*3-*R*-*T*_IDP1__–_*P_TEF1_-RofumR-T_RPL15A_*_–_*P*_TDH__3_*-TbFRDg-R-T_CYC1_*	This study
*UBR2*_CBS_-DHA-SA-*An*DCT-02	*MAT*α; *ubr2:UBR2_CBS 6412–13A_*; *gut1*:*P*_TEF1_-*Opgdh*-*T*_CYC1_-*P*_ACT1_-*DAK1*-*T*_TPS1_-*P*_PGK1_-*CjFPS1*-*T*_RPL15A_-*P*_TDH3_-*DAK1*-*T*_IDP1_; YGLCτ3:*P*_PGK1_-*MDH*3-*R*-*T*_IDP1__–_*P_TEF1_-RofumR-T_RPL15A_*_–_*P*_TDH__3_*-TbFRDg-R-T_CYC1_*; YPRCτ3:*P_ENO2_-An*DCT-02*-T_DIT1_*	This study
*UBR2*_CBS_-DHA-*An*DCT-02	*MAT*α; *ubr2:UBR2_CBS 6412–13A_*; *gut1*:*P*_TEF1_-*Opgdh*-*T*_CYC1_-*P*_ACT1_-*DAK1*-*T*_TPS1_-*P*_PGK1_-*CjFPS1*-*T*_RPL15A_-*P*_TDH3_-*DAK1*-*T*_IDP1_; YPRCτ3:*P_ENO2_-An*DCT-02-*T*_DIT1_	This study
*UBR2*_CBS_-DHA-*An*DCT-02 *icl1*Δ	*MAT*α; *ubr2:UBR2_CBS 6412–13A_*; *gut1*:*P*_TEF1_-*Opgdh*-*T*_CYC1_-*P*_ACT1_-*DAK1*-*T*_TPS1_-*P*_PGK1_-*CjFPS1*-*T*_RPL15A_-*P*_TDH3_-*DAK1*-*T*_IDP1_; YPRCτ3:*P_ENO2_-An*DCT-02-*T*_DIT1_; *icl1*:*loxP-ble-loxP*	This study
*UBR2*_CBS_-DHA-SA-*An*DCT-02 *icl1*Δ	*MAT*α; *ubr2:UBR2_CBS 6412–13A_*; *gut1*:*P*_TEF1_-*Opgdh*-*T*_CYC1_-*P*_ACT1_-*DAK1*-*T*_TPS1_-*P*_PGK1_-*CjFPS1*-*T*_RPL15A_-*P*_TDH3_-*DAK1*-*T*_IDP1_; YGLCτ3:*P*_PGK1_-*MDH*3-*R*-*T*_IDP1__–_*P_TEF1_-RofumR-T_RPL15A_*_–_*P*_TDH__3_*-TbFRDg-R-T_CYC1_*; YPRCτ3:*P_ENO2_-An*DCT-02-*T*_DIT1_; *icl1*:*loxP-ble-loxP*	This study
*UBR2*_CBS_-L-G3P	*MAT*α; *ubr2:UBR2_CBS 6412–13A_*; YGLCτ3*:P_TEF1_-CjFPS1-T_CYC1_*	Swinnen et al., 2016
*UBR2*_CBS_-L-G3P-SA-*An*DCT-02	*MAT*α*; ubr2:UBR2_CBS 6412–13A_*; YGLCτ3*:P_TEF1_-CjFPS1-T_CYC1_*; YPRCτ3:*P*_PGK1_-*MDH*3-*R*-*T*_IDP1__–_*P_TEF1_-RofumR-T_RPL15A_*_–_*P*_TDH__3_*-TbFRDg-R-T_CYC1_*-*P_ENO2_-An*DCT-02-*T*_DIT_	This study

### General Molecular Biology Techniques

Preparative PCRs for cloning as well as for sequence determination of expression cassettes integrated in the genome were performed using Phusion^®^ High-Fidelity DNA Polymerase (New England BioLabs, Frankfurt am Main, Germany). PCR conditions were adapted to the guidelines of the respective manufacturer. Restriction enzymes, FastAP alkaline phosphatase and T4 DNA ligase were obtained from Thermo Fisher Scientific (Waltham, MA, United States) and used according to the manufacturer’s instructions. PCR products were purified by using the GeneJET PCR Purification Kit (Thermo Fischer Scientific) and DNA fragments obtained after restriction were excised and purified using the QIAquick Gel Purification Kit (Qiagen, Hilden, Germany). Transformation of *S. cerevisiae* with plasmids as well as linear expression cassettes for genomic integration was performed according to the lithium acetate method described by Gietz et al. (1995).

### Construction of Expression Cassettes for Genomic Integration

The plasmids used within this study are listed in Supplementary Table S1. Codon-optimized coding sequences for *S. cerevisiae MDH3*, *Rhizopus oryzae fumR*, *Trypanosoma brucei FRDg*, and *Aspergillus niger* DCT-02, were kindly provided by Royal DSM N.V. (Delft, Netherlands). Mdh3 and Frd were targeted to the cytosol by removal of the peroxisomal targeting signal (SKL) from the protein, hereafter referred to as *MDH3*-*R* and *TbFRDg*-*R*, respectively (van de Graaf et al., 2015, Patent No. US20150057425A1). The cassettes for expression of *MDH3-R* under the control of the *PGK1* promoter and the *IDP1* terminator, *RofumR* under the control of the *TEF1* promoter and the *RPL15A* terminator, *TbFRDg-R* under the control of the *TDH3* promoter and the *CYC1* terminator, and *An*DCT-02 under the control of the *ENO2* promoter and the *DIT1* terminator, were assembled in pUC18 using Gibson isothermal assembly (Gibson et al., 2009). All primers used for amplification of the respective promoters, coding sequences and terminators are listed in Supplementary Table S2. All promoters and terminators were amplified from genomic DNA isolated from the *S. cerevisiae* strain S288C. One-step isothermal DNA assembly reactions contained 15 μL of the reagent-enzyme mix as described by Gibson et al. (2009), 0.05 pmol of *Bam*HI linearized pUC18 and threefold excess of the inserts [promoter, coding sequence, and the terminator (each 0.15 pmol)] in a final volume of 20 μL. Reaction mixtures were incubated at 50°C for 1 h and subsequently 5 μL of the reaction were directly used for transformation of *E. coli* DH5α. The resulting vectors were named pUC18-*MDH3-R*, pUC18-*RofumR*, pUC18-*TbFRDg-R*, and pUC18-*An*DCT-02 (Supplementary Table S1).

### Plasmids for CRISPR-Cas9 Mediated Genome Editing in *S. cerevisiae*

For CRISPR-Cas9 mediated genome editing, the vectors p414-TEF1p-Cas9-CYC1t-nat1 (Klein et al., 2016), p426-SNR52p-gRNA.YGLCτ3-SUP4t*-*hphMX (Islam et al., 2017) and p426-SNR52p-gRNA.YPRCτ3-SUP4t-hphMX were used (Supplementary Table S1). The vector p426-SNR52p-gRNA.YPRCτ3-SUP4t-hphMX was constructed from p426-SNR52p-gRNA.CAN1.Y-SUP4t-hphMX (Klein et al., 2016) by exchanging the 20 nt sequence at the 5′ end of the expressed gRNA targeting the *S. cerevisiae CAN1* gene by a 20 nt sequence targeting the long terminal repeat YPRCτ3 on chromosome XVI (Flagfeldt et al., 2009). The new target sequence was inserted by generating two overlapping PCR products of the gRNA expression cassette and their subsequent assembly in the same vector backbone according to Gibson et al. (2009). The CRISPRdirect online tool developed by Naito et al. (2015) was used to select the target sequence within the YPRCτ3 locus. Primers 463 and 596 and 460 and 597 (Supplementary Table S2) were used to amplify the overlapping fragments of the gRNA expression cassette from p426-SNR52p-gRNA.CAN1.Y-SUP4t-hphMX. Primers 460 and 463 generated overlaps to the ends of the *Pvu*II linearized p426-SNR52p-gRNA.CAN1.Y-SUP4t-hphMX while primers 596 and 597 generated the overlaps containing the YPRCτ3 targeting sequence. The *Pvu*II linearized p426-SNR52p-gRNA.CAN1.Y-SUP4t-hphMX was gel-purified after restriction. Gibson isothermal assembly was done as described in section “Construction of Expression Cassettes For Genomic Integration” yielding p426-SNR52p-gRNA.YPRCτ3-SUP4t-hphMX.

### *S. cerevisiae* Strain Construction

#### General Strategies for Genomic Integrations by Employing the CRISPR-Cas9 System

For genomic integrations the long terminal repeats YGLCτ3 and YPRCτ3 on chromosomes VII and XVI were used (Flagfeldt et al., 2009). Expression of Cas9 in the desired strain was achieved by transformation with the plasmid p414-TEF1p-Cas9-CYC1t-nat1 (Supplementary Table S1). In all strains the “SA module” was integrated at the YGLCτ3 locus (with the help of gRNA expression from p426-SNR52p-gRNA.YGLCτ3-SUP4t*-*hphMX). The expression cassette for *An*DCT-02 was integrated either at the YPRCτ3 locus (using p426-SNR52p-gRNA.YPRCτ3-SUP4t-hphMX for gRNA expression) in strains *UBR2*_CBS_-DHA-*An*DCT-02 and *UBR2*_CBS_-DHA-SA-*An*DCT-02 or together with the “SA module” at the YGLCτ3 locus in strain *UBR2*_CBS_-L-G3P-SA-*An*DCT-02. The expression cassettes were PCR-amplified from the plasmids carrying the respective expression cassettes using the primer pairs listed in Supplementary Table S2. These primers contained 5′-extensions generating 40–60 bp sequences homologous to regions directly upstream and downstream of the inserted double strand break at the integration site or to the respective adjacent expression cassette (in case several cassettes were assembled at the same locus). Co-transformation of the *S. cerevisiae* strain expressing the Cas9 endonuclease with the expression cassettes and the respective vector for gRNA expression resulted in assembly and integration of all expression cassettes at the target locus. Positive transformants were selected on YPD agar containing both nourseothricin and hygromycin B. Both vectors were subsequently removed from the resulting clone by serial transfers in YPD medium lacking the respective antibiotics yielding the desired strain. Subsequently, all integrated expression cassettes were sequenced.

The deletion of *ICL1* was obtained using a disruption cassette consisting of the phleomycin resistance marker (*ble*^r^) flanked by regions complementary to the upstream and downstream regions of the respective gene to be deleted. The deletion cassette was amplified from plasmid pUG66 (Supplementary Table S1) using primers 1243 and 1244 (Supplementary Table S2). The primers contained at their 5′ terminal end a 60 bp sequence complementary to the region immediately upstream or downstream of the start or stop codon of the gene to be deleted.

#### Strain SA

The expression cassettes for *MDH3-R*, *RofumR* and *TbFRDg-R* were PCR-amplified from the plasmids pUC18-*MDH3-R*, pUC18-*RofumR*, and pUC18-*TbFRDg-R* using primers pairs 690/770, 771/590, and 591/772, respectively (Supplementary Tables S1, S2). Subsequently, they were integrated at the YGLCτ3 locus of strain CEN.PK113-1A (*MATa*), by employing the CRISPR-Cas9 system, yielding strain SA. Strain CEN.PK113-1A (*MATa*) was obtained by switching the mating type of strain CEN.PK113-1A from *MATα* to *MATα* using the same strategy as described in Ho et al. (2017).

#### Strain *UBR2*_CBS_-DHA-SA

In order to bring the “SA module” into strain *UBR2*_CBS_-DHA (CEN.PK 113-1A *UBR2_CBS 6412–13A_ gut1:Opgdh-DAK1-CjFPS1-DAK1_OE–__2_* in Klein et al., 2016), the two strains were mated as shown in Supplementary Figure S1. This strategy was used to avoid homologous recombination events between the promoters and terminators, which were used for the generation of the expression cassettes to be integrated, with those already present in strain *UBR2*_CBS_-DHA upon transformation.

Mating, sporulation, and tetrad analysis were performed by procedures described by Sherman and Hicks (1991). Tetrads were dissected by using the micromanipulator from Singer Instruments Co., Ltd. (Roadwater Watchet Somerset, United Kingdom). Mating types were determined by diagnostic PCR for the *MAT* locus (Huxley et al., 1990). Mating type PCR was performed to verify haploidy of the obtained segregants while the presence of the “DHA” and “SA modules” was verified by diagnostic PCR and sequencing. Afterward, the *UBR2* in the respective haploid strain was sequenced to verify that the haploid strains contained the *UBR2* allele from CBS 6412-13A (*UBR*_CB__S_) (Swinnen et al., 2016).

#### Strains *UBR2*_CBS_-DHA-*An*DCT-02 and *UBR2*_CBS_-DHA-SA-*An*DCT-02

The expression cassette for *An*DCT-02 was PCR-amplified from the plasmid pUC18-*An*DCT-02 using primers pair 871/872 (Supplementary Tables S1, S2). The cassette was then integrated at the YPRCτ3 locus of strains *UBR2*_CBS_-DHA and *UBR2*_CBS_-DHA-SA, respectively, by employing the CRISPR-Cas9 system as described above, yielding strains *UBR2*_CBS_-DHA-*An*DCT-02 and *UBR2*_CBS_-DHA-SA-*An*DCT-02.

#### Strain *UBR2*_CBS_-L-G3P-SA-*An*DCT-02

The three expression cassettes (*MDH3-R*, *RofumR*, and *TbFRDg-R*) of the “SA module” and the expression cassette for *An*DCT-02 were amplified from the plasmids pUC18-*MDH3-R*, pUC18-*RofumR*, pUC18-*TbFRDg-R* and pUC18-*An*DCT-02 using primers pairs 991/770, 771/590, 591/992, and 993/872, respectively (Supplementary Tables S1, S2). All four expression cassettes for the “SA module” and *An*DCT-02 were integrated at the YGLCτ3 locus of the previously generated strain CEN.PK113-1A *UBR2_CBS_ CjFPS1* (Table 1; Swinnen et al., 2016) by employing the CRISPR-Cas9 system as described above, resulting in strain *UBR2*_CBS_-L-G3P -SA-*An*DCT-02.

### Isolation of Genomic DNA From *S. cerevisiae* Transformants and Diagnostic PCR

Correct integration of all expression and disruption cassettes was verified by diagnostic PCR using OneTaq Quick-load DNA polymerase and buffer according to the manufacturer’s guidelines (New England Biolabs, United Kingdom). Genomic DNA was isolated according to a modified protocol from Hoffman and Winston (1987). Single colonies obtained after transformations were re-streaked on respective agar plates. Approximately 50 mg of cells from these plates were suspended in 200 μL of TE buffer (10 mM Tris, 1 mM EDTA, pH 8.0). Subsequently, 300 mg of acid-washed glass beads (diameter of 0.425–0.6 mm) and 200 μL of phenol:chloroform:isoamyl alcohol (25:24:1) were added. The tubes were vortexed at maximum speed for 2 min and centrifuged at 15,700 g for 10 min. The aqueous phase (1 μL) was used as template in 20 μL PCR reactions. PCR primers were designed to bind upstream and downstream of the genomic integration sites as well as within the integrated expression/deletion cassette. For analysing integrations of multiple expression cassettes, additional primers were designed to produce amplicons covering the junctions between the individual integrated expression cassettes. After each integration step, the presence of all previously integrated expression cassettes was verified.

### Media and Cultivation Conditions for the Production of SA From Glycerol

All pre-cultures were cultured in synthetic medium-containing 20 g/L glucose and ammonium sulfate as the carbon and nitrogen source, respectively. All experiments for assessing SA production in shake flask batch cultivation were performed in synthetic medium, containing 60 mL/L (75.6 g/L) glycerol as the sole carbon source with urea as the nitrogen source. The synthetic medium was prepared according to Verduyn et al. (1992) containing 3 g/L KH_2_PO_4_, 0.5 g/L MgSO_4_.7H_2_O, 15 mg/L EDTA, 4.5 mg/L ZnSO_4_.7H_2_O, 0.84 mg/L MnCl_2_.2H_2_O, 0.3 mg/L CoCl_2_.6H_2_O, 0.3 mg/L CuSO_4_.5H_2_O, 0.4 mg/L NaMoO_4_.2H_2_O, 4.5 mg/L CaCl_2_.2H_2_O, 3 mg/L FeSO_4_.7H_2_O, 1 mg/L H_3_BO_3_, and 0.1 mg/L KI. After heat sterilization of the medium, filter sterilized vitamins were added. Final vitamin concentrations were: 0.05 mg/L D-(+)-biotin, 1 mg/L D-pantothenic acid hemicalcium salt, 1 mg/L nicotinic acid, 25 mg/L myo-inositol, 1 mg/L thiamine chloride hydrochloride, 1 mg/L pyridoxine hydrochloride, and 0.2 mg/L 4-aminobenzoic acid. In case urea was used as the nitrogen source (in main culture media), an appropriate aliquot of a stock solution was added after autoclaving to obtain a final concentration of 2.8 g/L while in all other cultures 5 g/L ammonium sulfate was added (in pre-culture media) before heat sterilization. The pH of the synthetic glucose medium was adjusted to 6.5 with 4 M KOH, while that of synthetic glycerol medium was adjusted to 4.0 with 2 M H_3_PO_4_.

For pre-cultivation, cells from a single colony were used to inoculate 3 mL of the synthetic glucose medium in a 10 mL glass tube and incubated at orbital shaking of 200 rpm and 30°C for 16 h. The pre-culture was used to inoculate 10 mL of the same medium in a 100 mL Erlenmeyer flask adjusting an OD_600_ of 0.2. This culture, hereafter referred to as intermediate culture, was cultivated at the same conditions for 48 h. The appropriate culture volume from the intermediate culture (in order to later adjust an OD_600_ of 0.2 in 100 mL of synthetic glycerol medium) was centrifuged at 800 g for 5 min and the supernatant discarded. The cell pellet was then washed once by re-suspending the cells in synthetic glycerol medium. The cell suspension was centrifuged again and re-suspended in 100 mL of the same medium in a 500 mL Erlenmeyer flask. The main cultures were incubated at orbital shaking of 200 rpm and 30°C and samples for OD_600_ determination and HPLC analysis were taken at regular time intervals. The culture biomass for strain *UBR2*_CBS_-DHA was determined by correlating OD_600_ measurements to dry weight. As a representative for determining the correlation, this strain was used.

### Metabolite Analysis by HPLC

Samples of culture supernatants (1 mL) were first filtered through 0.2 μm Minisart RC membrane filters (Sartorius, Göttingen, Germany) and if required stored at −20°C until analysis. The concentrations of SA, glycerol and ethanol in culture media were determined using a Waters HPLC system (Eschborn, Germany) consisting of a binary pump system (Waters 1525), injector system (Waters 2707), the Waters column heater module WAT038040, a refractive index (RI) detector (Waters 2414) and a dual wavelength absorbance detector (Waters 2487). The samples were injected onto an Aminex HPX-87H cation exchange column (Biorad, München, Germany) coupled to a Micro-guard^®^ column (Biorad) and eluted with 5 mM H_2_SO_4_ as the mobile phase at a flow rate of 0.6 mL/min and a column temperature of 45°C. Volumes of 20 μL of sample were used for injection. SA was detected using the dual wavelength absorbance detector (Waters 2487) while ethanol and glycerol were analyzed with the RI detector (Waters 2414). The retention time for SA was 11.2 min, for glycerol 13.5 min and for ethanol 22.7 min. Data were processed and analyzed using the Breeze 2 software (Waters).

### Bioreactor Experiments

For each bioreactor experiment cells from a single colony were used to inoculate 100 mL synthetic medium containing 75.6 g/L glycerol (prepared as described in section “Media and Cultivation Conditions for the Production of SA From Glycerol” but instead of urea, ammonium sulfate was used as nitrogen source) in 500 mL round-bottom shake flasks and incubated in an Innova 44 incubator (New Brunswick Scientific, Edison, NJ, United States) set at 30°C and 200 rpm overnight. Cells growing exponentially in fresh medium were then used to inoculate the bioreactors at an initial OD of 1.0, measured at 600 nm in a Libra S12 spectrophotometer (Biochrom, Cambridge, United Kingdom). Aerobic batch cultures were grown in 2-L bioreactors (Applikon, Delft, Netherlands) at a starting working volume of 1 L synthetic glycerol medium with ammonium sulfate (5 g/L) as nitrogen source. In order to sustain higher biomass concentrations, the vitamin and trace elements concentrations were increased (twofold) and additional biotin was added separately to reach a final medium concentration of 1 mg/L as described by Wahl et al. (2017). The pH of the culture was maintained at 5.0 via automated addition of 10 M KOH and the temperature was maintained at 30°C. The inflowing gas was an in-line mix of pressurized air and pure carbon dioxide (>99.7% purity, Linde Gas Benelux, Schiedam, Netherlands) at a combined flowrate of 500 mL/min controlled with mass flow controllers (Brooks, Hatfield, PA, United States). The resulting gas mixture contained 10.3% carbon dioxide and 18.6% oxygen. The bioreactor was stirred at 800 rpm. Samples for OD_600_ determination and HPLC analysis were taken at regular time intervals.

### Off-Gas Analysis, Biomass, and Extracellular Metabolite Determinations

The exhaust gas from the bioreactor was cooled with a condenser at 2°C and dried with a PermaPure Dryer (model MD 110-8P-4; Inacom Instruments, Veenendaal, Netherlands). The dried gas was analyzed for its carbon dioxide and oxygen concentration with a combined paramagnetic/infrared off-gas analyzer NGA2000 Analyzer (Rosemount, Baar, Switzerland). The biomass concentrations were determined by filtering duplicate, exact volumes of culture broth (5 mL), over pre-dried Supor 47 membrane filters with a 0.45 μm pore size, washed and then dried in a microwave oven for 20 min at 360 W and weighed again (Pall Laboratory, Port Washington, NY, United States) as described by Postma et al. (1989). Extracellular metabolites of the cultures were obtained by centrifuging culture samples (10 min at 3500 g) of which the supernatant was analyzed by HPLC as described previously by Hakkaart et al. (2020).

## Results

### Establishing the Genetic Modifications Aiming at Reductive SA Production and Its Export in a *S. cerevisiae* Strain Equipped With the DHA Pathway

A *S. cerevisiae* CEN.PK113-1A derivative in which the native FAD-dependent L-G3P pathway for glycerol catabolism had been replaced by the NAD-dependent DHA pathway (Klein et al., 2016) was used as a baseline strain. Apart from the genetic modifications previously referred to as “DHA pathway module II,” this strain carried a replacement of the endogenous *UBR2* allele by the respective allele from the glycerol-utilizing wild-type isolate CBS 6412-13A (*UBR2*_CB__S_) as described by Swinnen et al. (2016). The latter modification proved to be crucial for the “DHA pathway strain” to achieve the reported maximum specific growth rate (μ*_*max*_*) of ∼0.26 h^–1^ in synthetic glycerol medium (Klein et al., 2016). In the current study, the used baseline strain is referred to as strain *UBR2*_CBS_-DHA (Table 1). The DHA pathway module (“DHA module”) consists of all genetic modifications required for the expression of an NAD-dependent glycerol dehydrogenase from *Ogataea parapolymorpha* (*Op*gdh), the overexpression of endogenous *DAK1* (two expression cassettes, each under the control of a different promoter) as well as the expression of the aquaglyceroporin Fps1 from *Cyberlindnera jadinii* (*Cj*Fps1) to increase the glycerol uptake rate. In the described strain the L-G3P pathway is effectively abolished as all four expression cassettes had been integrated at the *GUT1* (Klein et al., 2016).

The strain *UBR2*_CBS_-DHA was used in order to establish the cytosolic reductive SA pathway from oxaloacetate to SA, using the strategy employed by van de Graaf et al. (2015). In detail, we (over)expressed the endogenous peroxisomal malate dehydrogenase (*MDH3*), which is responsible for oxaloacetate reduction, the heterologous cytosolic fumarase (*fumR*) from *Rhizopus oryzae* for conversion of malate to fumarate and the peroxisomal fumarate reductase (*FRD*g) from *Trypanosoma brucei* for fumarate reduction (Figure 2). For targeting of Mdh3 and Frd to the cytosol, the peroxisomal targeting signals (SKL) were removed from the proteins (the respective genes are referred to here as *MDH3*-*R* and *TbFRDg-R*). The aforementioned genetic modifications are collectively referred to as “SA module” throughout this study (Figure 2) and the resulting strain was named *UBR2*_CBS_-DHA-SA. Subsequently, we inserted an expression cassette for the dicarboxylic acid transporter encoded by *An*DCT-02 resulting in the strain *UBR2*_CBS_-DHA-SA-*An*DCT-02 (see section “Materials and Methods” for details).

In an attempt to scrutinize the effect of the sole expression of the transporter *An*DCT-02 (without the “SA module”), the strain *UBR2*_CBS_-DHA-*An*DCT-02 was constructed. Moreover, *icl1* deletion mutants were constructed for strains *UBR2*_CBS_-DHA-*An*DCT-02 and *UBR2*_CBS_-DHA-SA-*An*DCT-02. *ICL1* encodes isocitrate lyase, which is one of the two key enzymes of the glyoxylate cycle (Figure 2) and the mutants were used in order to test the contribution of the endogenous glyoxylate cycle on SA formation of the respective strains. It is worth mentioning that a previous study in our lab has shown that abolishment of the glyoxylate cycle (deletion of *ICL1*) had no significant effect on growth of our baseline strain *UBR2*_CBS_-DHA in synthetic glycerol medium (Xiberras et al., 2020). However, this result does not completely exclude that carbon is channeled via the glyoxylate cycle.

### The Enzymes of Both the “SA Module” and the Endogenous Glyoxylate Cycle Contribute to SA Production in a Synergistic Manner

The strains *UBR2*_CBS_-DHA, *UBR2*_CBS_-DHA-*An*DCT-02, *UBR2*_CBS_-DHA-SA-*An*DCT-02 as well as the *icl1* deletion mutants corresponding to the latter two strains were first tested in shake flask experiments using synthetic glycerol medium. In contrast to the study of Klein et al. (2016), urea was used as a nitrogen source instead of ammonium sulfate in order to minimize further acidification and weak acid stress (de Kok et al., 2012). We used 100 mL medium in 500 mL shake flasks. This setup has previously been shown to induce some alcoholic fermentation in *S. cerevisiae* DHA pathway strains (Aßkamp et al., 2019). The idea was to also provide conditions here in which respiratory metabolism is limited to increase cytosolic NADH availability and facilitate the flux to SA in strains equipped with the redox-neutral pathway.

**FIGURE 3:**
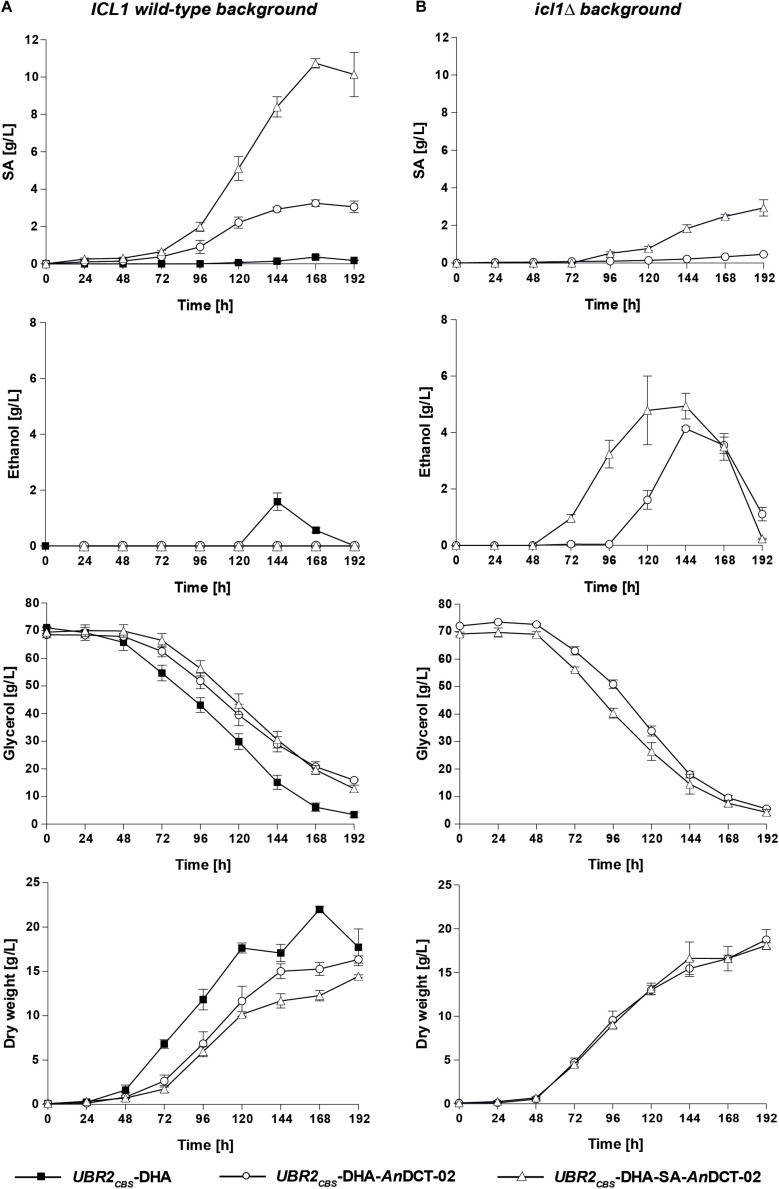
Production of SA, ethanol and biomass as well as consumption of glycerol in shake flask experiments with the *S. cerevisiae* strains *UBR2*_CBS_-DHA, *UBR2*_CBS_-DHA-*An*DCT-02, and *UBR2*_CBS_-DHA-SA-*An*DCT-02 **(A)** and the strains *UBR2*_CBS_-DHA-*An*DCT-02 *icl1*Δ and *UBR2*_CBS_-DHA-SA-*An*DCT-02 *icl1*Δ **(B)**. Cultivations were performed in 500 mL flasks containing 100 mL of synthetic medium containing 75.6 g/L glycerol as the sole carbon source and culture supernatants were analyzed by HPLC for production of SA, ethanol, and the consumption of glycerol. Growth was recorded by optical density measurements at 600 nm. The culture biomass was determined by correlating OD_600_ measurements to dry weight as described in section “Materials and Methods.” Mean values and standard deviations were determined from at least three biological replicates.

As expected, only marginal amounts of SA could be detected for the baseline strain *UBR2*_CBS_-DHA (Figure 3A – *ICL1* wild-type background). However, up to 1.6 g/L of the natural fermentation product ethanol was detected in the culture supernatant. Interestingly, the sole expression of *An*DCT-02 in *UBR2*_CBS_-DHA had a significant effect on the physiology of the strain. It resulted in a decreased specific growth rate and glycerol consumption rate and the formation of 3.2 g/L SA after 168 h of cultivation without detectable ethanol production (Figure 3A). This result implies that the *An*DCT-02 transporter was able to even facilitate significant excretion of the SA solely formed via endogenous pathways.

The additional expression of the “SA module” in the cytosol of strain *UBR2*_CBS_-DHA-*An*DCT-02 led to a remarkable increase in product titer. The maximum titer was 10.7 g/L after 168 h which corresponds to a yield of 0.22 ± 0.01 g SA/g glycerol. Notably, the sole presence of the “SA module” in strain *UBR2*_CBS_-DHA (without *An*DCT-02) resulted in a significant improvement of growth compared to the baseline strain, but only marginal SA production (1.6 g/L; data not shown).

SA is an intermediate of the glyoxylate cycle and it is known that the key enzymes of this pathway (isocitrate lyase and malate synthase) are highly upregulated when *S. cerevisiae* is grown in medium containing glycerol as the sole carbon source, i.e., mRNA levels were significantly increased compared to those obtained from cells growing in glucose-containing medium (Roberts and Hudson, 2006). It was therefore unclear whether the SA formed in the strains *UBR2*_CBS_-DHA-*An*DCT-02 and *UBR2*_CBS_-DHA-SA-*An*DCT-02 indeed resulted from the established reductive carbon dioxide fixing pathway. We deleted *ICL1* in the respective strains in order to get an indication about the contribution of the glyoxylate cycle. The respective deletions resulted in the complete abolishment and substantial reduction of SA formation in *UBR2*_CBS_-DHA-*An*DCT-02 and *UBR2*_CBS_-DHA-SA-*An*DCT-02, respectively (Figures 3A,B). Interestingly, growth and glycerol consumption were not negatively affected but even slightly improved by the *ICL1* deletion (Figures 3A,B). However, ethanol (up to 4.9 g/L) became the predominant fermentation product in these mutants (Figure 3B). While there was an interim increase of the pH from 4 to 6 for strains *UBR2*_CBS_-DHA, *UBR2*_CBS_-DHA-*An*DCT-02 *icl1*Δ and *UBR2*_CBS_-DHA-SA-*An*DCT-02 *icl1*Δ (Supplementary Figure S2), the production of SA seemed to have counteracted this pH increase since it was much less pronounced in the SA producing strains *UBR2*_CBS_-DHA-*An*DCT-02 and *UBR2*_CBS_-DHA-SA-*An*DCT-02.

The considerable reduction of SA production caused by abolishing the activity of the key enzyme of the glyoxylate cycle Icl1 suggests that a major part of SA in the strain *UBR2*_CBS_-DHA-SA-*An*DCT-02 included carbon flux via the latter pathway (Figure 2). In fact, only the combined activity of both the “SA module” and the endogenous route via the PDH bypass and the glyoxylate cycle (see Figure 2) enabled the highest SA production (10.7 g/L).

We also checked to which extent the genetic modifications of the “DHA module” (replacement of the native FAD-dependent L-G3P pathway by the NADH-delivering DHA pathway and the presence of the heterologous Fps1) promoted SA overproduction in the genetic background of the strain *UBR2*_CBS_-DHA-SA-*An*DCT-02. For this purpose, a corresponding CEN.PK113-1A derivative was constructed which catabolized glycerol via the L-G3P pathway but also contained the “SA module” and *An*DCT-02 (strain *UBR2*_CBS_-L-G3P-SA-*An*DCT-02, see section “Materials and Methods”). The latter strain showed a strongly decreased SA formation (from 10.7 to 3.2 g/L) compared to the isogenic strain with the “DHA module” while growth and glycerol consumption were only slightly reduced (Supplementary Figure S3). In fact, SA titers in strain *UBR2*_CBS_-L-G3P-SA-*An*DCT-02 were reduced to levels similar to those observed in strain *UBR2*_CBS_-DHA-*An*DCT-02 (with “DHA module” but without “SA module,” Figure 3) suggesting that the surplus of cytosolic NADH provided by the DHA pathway is indeed crucial for the observed high SA levels in *UBR2*_CBS_-DHA-SA-*An*DCT-02.

### Characterization of Strain *UBR2*_CBS_-DHA-SA-*An*DCT-02 in Bioreactors

**FIGURE 4:**
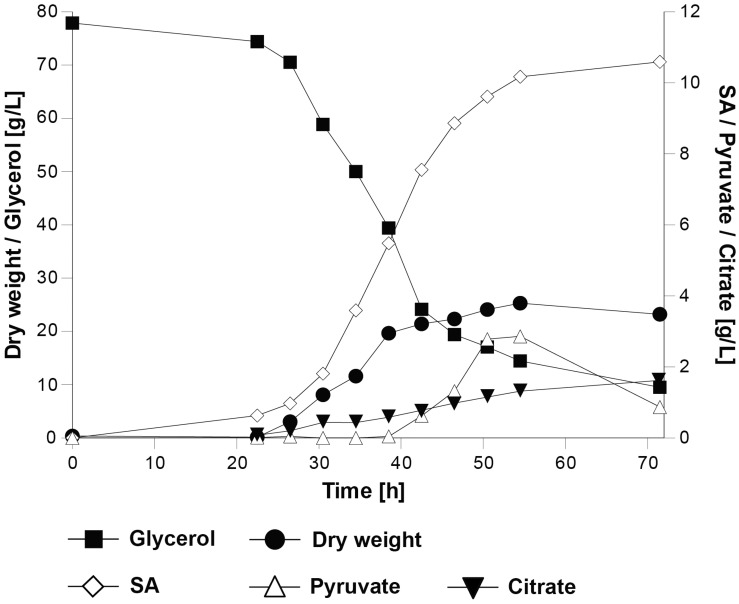
Physiological characterization of the *S. cerevisiae* strain *UBR2*_CBS_-DHA-SA-*An*DCT-02 in batch cultivations in 2-L bioreactors using synthetic medium containing 75.6 g/L glycerol as the carbon source and ammonium sulfate as nitrogen source (pH was kept at a level of 5.0 throughout the cultivation). One representative out of two experiments is shown.

We hypothesized that an increased availability of carbon dioxide in the culture medium could facilitate the metabolic flux through the reductive SA pathway. For this purpose, strain *UBR2*_CBS_-DHA-SA-*An*DCT-02 was characterized under controlled conditions in batch cultivations in bioreactors and sparged with 10.3% carbon dioxide at 500 mL/min. In contrast to the shake flask experiments, ammonium sulfate was used as the source of nitrogen; pH control (at pH 5.0) avoided acidification. The controlled cultivation conditions led to a higher specific growth rate and final biomass concentration (Figure 4) than in shake flasks (Figure 3A), which might be due to the better oxygen transfer rate, increased carbon dioxide availability and/or better pH control in the culture. The maximum SA titer of 10.3 g/L reached in the bioreactor experiments (Figure 4) was similar to that obtained in shake flask batch cultivations. The obtained SA yield (0.16 ± 0.00 g/g) was 28% lower than in the shake flask experiments. Interestingly, pyruvate and citric acid were detected as additional products only during the bioreactor cultivations but not in the shake flasks. Pyruvate accumulated after 55 h (up to 2 g/L), after which the titer decreased while citrate accumulated slowly during the entire course of the cultivation (up to ∼2 g/L). Calculation of a solid carbon balance at 71.5 h (time point at which the highest SA titer was detected) revealed that 16.4% of carbon from glycerol was found in SA, 1.4% in pyruvate, 2.3% in citrate, 43.2% in biomass and 32.7% in the formed carbon dioxide, which is equivalent to 96% recovery of the total carbon.

## Discussion

The production of high quantities of SA from glucose in the robust production host *S. cerevisiae* is state-of-the-art and has even already been commercially applied (Ahn et al., 2016). The targeted reductive pathway is highly attractive due to fixation of carbon dioxide (Steiger et al., 2017). As discussed in the introduction, the use of glycerol allows a higher theoretical SA yield compared to glucose. The initial goal of this study was to establish a redox-neutral pathway for the reductive production of SA from glycerol in one of our previously constructed glycerol-catabolizing “DHA pathway strains” of *S. cerevisiae*. The results demonstrate that it is possible to produce significant amounts of SA from glycerol-containing medium in shake flask experiments as soon as the strain is equipped with the “DHA module,” the “SA module” and the *An*DCT-02 transporter. In bioreactor experiments, carbon dioxide sparging seems to be crucial for significant SA production, since trial experiments without additional carbon dioxide resulted in significantly lower SA titers (data not shown). A possible explanation could be that the carboxylation of pyruvate was rate-controlling and enhanced by the higher carbon dioxide concentration.

One major question addressed in the current study was whether the SA was indeed produced via the targeted redox-neutral pathway or endogenous pathways at least contributed to product formation. In principle, SA can also be produced via the pyruvate dehydrogenase bypass followed by the glyoxylate cycle (Figure 2). Abolishing the flux through the glyoxylate cycle by deleting *ICL1* in the strain *UBR2*_CBS_-DHA-SA-*An*DCT-02 reduced the maximum SA concentration from 10.7 to 2.9 g/L. This led us to the assumption that the glyoxylate cycle had an important impact on SA production our engineered strains.

Nevertheless, the established “SA module” must also have significantly contributed to SA production in strain *UBR2*_CBS_-DHA-SA-*An*DCT-02 as the SA titer was much higher (10.7 g/L) compared to an isogenic strain that carries the *An*DCT-02 transporter but no “SA module” (3.2 g/L). Considering these results plus those obtained with the *icl1* mutant, we hypothesize a synergistic effect between the endogenous pyruvate dehydrogenase bypass/glyoxylate cycle route and the last two enzymes of the “SA module” (Figure 2). This hypothesis still has to be confirmed by metabolic flux analysis in the future.

Interestingly, the deletion of *ICL1* in strains *UBR2*_CBS_-DHA-*An*DCT-02 and *UBR2*_CBS_-DHA-SA-*An*DCT-02 caused an onset of ethanol formation and a slightly improved biomass formation compared to their respective *ICL1* wild-type counterparts. A slight ethanol formation was also visible in the baseline strain *UBR2*_CBS_-DHA. We assume that a partial switch to fermentation (which has never been observed in *S. cerevisiae* using the L-G3P pathway) is caused by the increased rate of NADH generation (DHA pathway) together with improved glycerol uptake (Aßkamp et al., 2019). A reduced oxygen transfer in shake flasks with filling volumes higher than the recommended 10% also seemed to have facilitated the ethanol production (Aßkamp et al., 2019). We also have good reasons to believe that the genetic strain background has a strong effect on the ethanol production/fermentation capacity of *S. cerevisiae* DHA pathway strains (unpublished results). The increased ethanol production in the *icl1* mutants could be explained by the fact that cytosolic acetyl-CoA resulting from the pyruvate dehydrogenase bypass cannot enter the glyoxylate cycle in the *icl1* deletion mutants. This might cause a redirection of carbon from acetaldehyde towards ethanol (Figure 2). The fact that carbon prefers to flow to ethanol rather than SA (in spite of a better redox balance in the latter scenario) is another indication that pyruvate carboxylation might be rate-controlling.

The improvement of biomass formation caused by the *ICL1* deletion is puzzling but could simply be caused by a significant reduction of SA production coupled to a reduced ATP dissipation during SA export and eventually a higher availability of ATP for biosynthesis processes. Additionally, cytosolic acetyl-CoA cannot enter the glyoxylate cycle in the *icl1* deletion mutants. The reduced conversion of acetate to acetyl-CoA by ACS (an ATP-dependent reaction) would also result in a higher availability of ATP (Figure 2).

As mentioned in the introduction, previously published studies aimed at the production of SA with *S. cerevisiae* via the cytosolic reductive SA pathway from glucose included an increased activity of pyruvate carboxylase. In fact, both Yan et al. (2014) and van de Graaf et al. (2015, Patent No. US20150057425A1) overexpressed the endogenous *PYC2* gene for this purpose. We initially did not include pyruvate carboxylase overexpression in our constructed strains since several studies indicated a significantly higher expression level of pyruvate carboxylase in wild-type *S. cerevisiae* growing on respiratory carbon sources compared to glucose (DeRisi et al., 1997; Menéndez and Gancedo, 1998; Huet et al., 2000; Dueñas-Sánchez et al., 2012). However, we later also overexpressed *PYC2* in strain *UBR2*_CBS_-DHA-SA-*An*DCT-02 and surprisingly observed a significant drop in SA titer accompanied by increased ethanol formation. We devoted a separate study in order to understand the significantly altered metabolic fluxes caused by *PYC2* overexpression (Xiberras et al., unpublished results).

The results obtained with the *PYC2* overexpression demonstrate our gaps in understanding the central metabolic fluxes when *S. cerevisiae* grows on glycerol as recently reviewed by Xiberras et al. (2019). One of our future goals is the application of ^13^C metabolic flux analysis to the constructed strains.

Another important genetic modification for SA production from glucose has been the abolishment of ethanol formation, since Yan et al. (2014) used a PDC-negative as the baseline strain (TAM strain) (van Maris et al., 2004). Most of our constructed strains did not produce significant amounts of ethanol when grown in glycerol-containing synthetic medium, which was in strong contrast to glucose-containing synthetic medium. In fact, strain *UBR2*_CBS_-DHA-SA-*An*DCT-02 was also characterized in synthetic medium containing 75.6 g/L glucose. As expected, the strain produced relatively high amounts of ethanol (maximum 30 g/L) from glucose but virtually no SA (data not shown). Apparently, the flux from acetaldehyde into the glyoxylate cycle was strong enough in glycerol-containing medium that most of the carbon was diverted away from ethanol production even without any genetic modification. Still, the abolishment of the glyoxylate cycle resulted in significant ethanol formation. Therefore, it might be that further strain engineering requires the prevention of ethanol formation.

The production of SA from glycerol and carbon dioxide via the envisaged pathway is not only redox- but also ATP neutral (Figure 2). However, one has to consider that the export energetics has major implications on the overall ATP yield and maximum product titers. Particularly in the case of weak acids, this can result in an upper limitation of the maximum theoretical SA yield in microbial fermentations, especially at low pH and high product titers. Although being an attractive approach for further improving SA production from glycerol and carbon dioxide, rational engineering of transport energetics remains challenging due to the insufficient knowledge regarding organic acid exporters in *S. cerevisiae* (Borodina, 2019).

Overall, the strains constructed here provide a starting point for further optimization of strain construction and process conditions for SA production from glycerol in the popular production host *S. cerevisiae*. Future work will focus on further metabolic engineering to overcome the rate-controlling steps of the pathway from oxaloacetae to SA, i.e., channel the carbon through the initially envisaged redox-neutral pathway. One challenge in strain engineering certainly results from the demand in ATP required for metabolite transport (as discussed) and pyruvate carboxylation. In fact, the current strain design requires part of the glycerol to be respired and/or fermented to ethanol to provide ATP for SA formation, cellular maintenance and growth, with a negative impact on the SA yield. Future studies could focus on these energetic requirements, by evaluating alternative transport systems and replacing the ATP-dependent pyruvate carboxylase by ATP-independent routes.

## Data Availability Statement

The datasets analyzed in this article are not publicly available. Requests to access the datasets should be directed to EN, e.nevoigt@jacobs-university.de.

## Author Contributions

JX, MK, and EN planned the study and wrote the manuscript. JX constructed all the strains and performed all the shake-flasks (batch) cultivations. JX, EH, and RM planned and performed the bioreactor experiment. EH and RM provided critical feedback to the manuscript.

## Conflict of Interest

Royal DSM N.V. provided us with the plasmids pGBS414PPK-3, pGBS415FUM-3, and pGBS416DCT-2 but was not involved in the study design, collection, analysis, interpretation of data, and writing of the article. The authors declare that the research was conducted in the absence of any commercial or financial relationships that could be construed as a potential conflict of interest.
