# Supplementary material for: Engineering Saccharomyces cerevisiae for Succinic Acid Production From Glycerol and Carbon Dioxide
Source: Front Bioeng Biotechnol. 2020 Jun 26;8:566. doi: 10.3389/fbioe.2020.00566 (PMC7332542; doi:10.3389/fbioe.2020.00566)
Supplement: Supplementary file 1 [file Data_Sheet_1.pdf]

Supplementary material

Table S1: Plasmids used in this study.

Plasmid	Relevant characteristic	Source or reference
**pGBS414PPK-3**	Codon-optimized coding sequence for glycosomal fumarate reductase from *T. brucei* lacking the codons for the peroxisomal targeting signal (*FRDg-R*)	DSM, The Netherlands
**pGBS415FUM-3**	Codon-optimized coding sequence for fumarase (*fumR*) from *R. oryzae* and peroxisomal malate dehydrogenase from *S. cerevisiae* (*MDH3-R*; removal of the codons for the peroxisomal targeting signal retargets the enzyme to the cytosol)	DSM, The Netherlands
**pGBS416DCT-02**	Codon-optimized coding sequence for the dicarboxylic acid transporter (DCT-02) from *A. niger*	DSM, The Netherlands
**p414-TEF1p-Cas9-CYCt-nat1**	*natMX4,* P_{TEF1}-*cas9*-T_{CYC1}	Klein et al. (2016)
**p426-SNR52p-gRNA.CAN1.Y-SUP4t-hphMX**	2 µm, *hphMX, SNR52p-gRNA.*CAN1.Y-*SUP4t*	Klein et al. (2016)
**p426-SNR52p-gRNA.YGLCτ3- SUP4t-hphMX**	2 µm, *hphMX, SNR52p-gRNA.*YGLCτ3-*SUP4t*	Islam et al. (2017)
**p426-SNR52p-gRNA.YPRCτ3-SUP4t-hphMX**	2 µm, *hphMX, SNR52p-gRNA.* YPRCτ3-*SUP4t*	This study
**pUG66**	ble^r	Gueldener et al. (2002)
**pUC18**	*E. coli* cloning vector	Yanisch-Perron et al. (1985)
**pUC18-*MDH3-R***	P_{PGK1}-*MDH3*-R-T_{IDP1}	This study
**pUC18-*RofumR***	P_{TEF1}-*RofumR*-T_{RPL15A}	This study
**pUC18-*TbFRDg-R***	P_{TDH3}- *TbFRDg-R*-T_{CYC1}	This study
**pUC18-*An*DCT-02**	P_{ENO2}-*An*DCT-*02*-T_{DIT1}	This study

Table S2: Primers used in this study.

Purpose	Primer number	Sequence (5'-3')	Description
**Exchange of the *CAN1* target sequence in the gRNA coding sequence of p426-SNR52p-gRNA.CAN1.Y-SUP4t-hphMX by a 20 nt sequence targeting YPRCτ3 region**	460	CCCGCGCGTTGGCCGATTCAT	Primer for Gibson Assembly - amplification of *SUP4* terminator and gRNA structural component with a 21 bp overhang homologous to the adjacent PvuII-linearized p426-SNR52p-gRNA.CAN1.Y-SUP4t-hphMX backbone
	597	GCTCTAAAACATACAGCGTTACCAATATGGGATCATTTATCTTTCACTGCGGAGAAG	Primer for Gibson Assembly - amplification of *SUP4* terminator and gRNA structural component containing the new 20 bp YPRCτ3 target sequence with a 35 bp overhang homologous to the adjacent SNR52 promoter of gRNA cassette
	596	ATAAATGATCCCATATTGGTAACGCTGTATGTTTTAGAGCTAGAAATAGCAAGTTAAAATAAGGC	Primer for Gibson Assembly - amplification of SNR52 promoter containing the new 20 bp YPRCτ3 target sequence with a 35 bp overhang homologous to the adjacent gRNA structural component
	463	GTCGACCTGCAGCGTACGAAGCTTCAG	Primer for Gibson Assembly - amplification of SNR52 promoter with a 16 bp overhang homologous to the adjacent PvuII-linearized p426-SNR52p-gRNA.CAN1.Y-SUP4t-hphMX backbone
**Construction of the expression cassette of *S. cerevisiae MDH3* (P_{PGK1}-*MDH3*-T_{IDP1}) in pUC18**	311	GCCAGTGCCAAGCTTGCATGCCTGCAGGTCGACTCTAGAGGATCCGAAGTACCTTCAAAGAATGGGGTCT	Primer for Gibson Assembly - amplification of *PGK1* promoter from *S. cerevisiae* Sc288c DNA containing a 40 bp overhang homologous to the adjacent BamHI-linearized-pUC18 vector backbone
	726	AAGATGGCAACCTTAACCATTGTTTTATATTTGTTGTAAAAAGTAGATAATTACTTCCTTGATGATCTG	Primer for Gibson Assembly - amplification of *PGK1* promoter from *S. cerevisiae* Sc288c containing a 30 bp overhang homologous to the adjacent *MDH3* open reading frame
	727	TTATCTACTTTTTACAACAAATATAAAACAATGGTTAAGGTTGCCATCTTAGGTG	Primer for Gibson Assembly - amplification of *MDH3* from pGBS415FUM-3 containing a 30 bp overhang homologous to the adjacent *PGK1* promoter
	728	AAAGTGGTAGATTGGGCTACGTAAATTCGATTAACTGTCCAAGATGAAAGACTTACCC	Primer for Gibson Assembly - amplification of *MDH3* from pGBS415FUM-3 with a 30 bp overhang homologous to the adjacent *IDP1* terminator
	729	AAGGGTAAGTCTTTCATCTTGGACAGTTAATCGAATTTACGTAGCCCAATC	Primer Gibson Assembly- amplification of *IDP1* terminator from *S. cerevisiae* Sc288c DNA with a 30 bp overhang homologous to the adjacent *MDH3* open reading frame
	576	GCTATGACCATGATTACGAATTCGAGCTCGGTACCCGGGGATGGTAATGATCCGAACTTGG	Primer Gibson Assembly - for amplification of *IDP1* terminator from *S. cerevisiae* Sc288c DNA with a 40 bp overhang homologous to the adjacent BamHI-linearized-pUC18 vector backbone

**Construction of the expression cassette of *R. oryzae fumR* (*P_{TEF1}-RfumR-T_{RPL15A}*) in pUC18**	410	TGCAGGTCGACTCTAGAGCATAGCTTCAAAATGTTTCTA CTCCTTTTTTACTCTTCC	Primer for Gibson Assembly - amplification of *TEF1* promoter from *S. cerevisiae* Sc288c DNA containing a 40 bp overhang homologous to the adjacent BamHI-linearized-pUC18 vector backbone
	730	GCAAAGCAGCAGAAGCAGAGGACATTTAGATTAGATT GCTATGCTTTCTTTCTAATGAGC	Primer for Gibson Assembly - amplification of *TEF1* promoter from *S. cerevisiae* Sc288c containing a 30 bp overhang homologous to the adjacent *RofumR* open reading frame
	731	TTAGAAAGAAAGCATAGCAATCTAATCTAAATGTCCTCT GCTTCTGCTG	Primer for Gibson Assembly - amplification of *RoFUMR* from pGBS415FUM-3 containing a 30 bp overhang homologous to the adjacent *TEF1* promoter
	732	ATAAAATTATATTTTCCATCAACCAGCTTATTAATCCTTG GCAGAAATCATGTCC	Primer for Gibson Assembly - amplification of *RoFUMR* from pGBS415FUM-3 with a 30 bp overhang homologous to the adjacent *RPL15A* terminator
	733	CCTGAGGACATGATTTCTGCCAAGGATTAATAAGCTGG TTGATGGAAAATATAATTTTATTGGGC	Primer Gibson Assembly- amplification of *RPL15A* terminator from *S. cerevisiae* Sc288c DNA with a 30 bp overhang homologous to the adjacent *RoFUMR* open reading frame
	354	GAAACAGCTATGACCATGATTACGAATTCGAGCTCGGT ACCCGGGGGAAAAACGGGAAGAAAAGGAAAGAAAAA AAATAC	Primer Gibson Assembly - for amplification of *RPL15A* terminator from *S. cerevisiae* Sc288c DNA with a 40 bp overhang homologous to the adjacent BamHI-linearized-pUC18 vector backbone
**Construction of the expression cassette of *T. brucei TbFRDg-R* (*P_{TDH3}-TbFRDg-R-T_{CYC1}*) in pUC18**	571	GCCAGTGCCAAGCTTGCATGCCTGCAGGTCGACTCTAG AGTCGAGTTTATCATTATCAATACTGC	Primer for Gibson Assembly - amplification of *TDH3* promoter from *S. cerevisiae* Sc288c DNA containing a 40 bp overhang homologous to the adjacent BamHI-linearized-pUC18 vector backbone
	721	ACAATGGAAGCAGAAGATCTACCATCAACCATCCGTCG AAACTAAGTTCTTG	Primer for Gibson Assembly - amplification of *TDH3* promoter from *S. cerevisiae* Sc288c containing a 30 bp overhang homologous to the adjacent *TbFRDg-R* open reading frame
	722	TAAAACACCAAGAACTTAGTTTCGACGGATGGTTGATG GTAGATCTTCTGC	Primer for Gibson Assembly - amplification of *TbFRDg-R* from pGBS414PPK-3 containing a 30 bp overhang homologous to the adjacent *TDH3* promoter
	723	AATGTAAGCGTGACATAACTAATTACATGATTAACTTCC AGATGGTTCAGTTTCG	Primer for Gibson Assembly - amplification of *TbFRDg-R* from pGBS414PPK-3 with a 30 bp overhang homologous to the adjacent *CYC1* terminator
	724	GTTGACGAAACTGAACCATCTGGAAGTTAATCATGTAA TTAGTTATGTCACGC	Primer Gibson Assembly- amplification of *CYC1* terminator from *S. cerevisiae* Sc288c DNA with a 30 bp overhang homologous to the adjacent *TbFRDg-R* open reading frame
	725	AGCTATGACCATGATTACGAATTCGAGCTCGGTACCCG GGGCAAATTAAAGCCTTCGAG	Primer Gibson Assembly - for amplification of *CYC1* terminator from *S. cerevisiae* Sc288c DNA with a 40 bp overhang homologous to the adjacent BamHI-linearized-pUC18 vector backbone

**Construction of the expression cassette of *A. niger* DCT-02 (*P_{ENO2}*-*An*DCT-02-*T_{DIT1}*) in pUC18**	716	GCCAGTGCCAAGCTTGCATGCCTGCAGGTCGACTCTAG AGGTGTCGACGCTGCGGGTA	Primer for Gibson Assembly - amplification of *ENO2* promoter from *S. cerevisiae* Sc288c DNA containing a 40 bp overhang homologous to the adjacent BamHI-linearized-pUC18 vector backbone
	717	AGAACCTGGCAAAGAAGTTTCAACGTTCATCATTATTAT TGTATGTTATAGTATTAGTTGCTTGGTG	Primer for Gibson Assembly - amplification of *ENO2* promoter from *S. cerevisiae* Sc288c containing a 30 bp overhang homologous to the adjacent *AnDCT02* open reading frame
	718	CAACTAATACTATAACATACAATAATAATGATGAACGTT GAAACTTCTTTGCCAG	Primer for Gibson Assembly - amplification of *An*DCT-02 from pGBS416DCT-2 containing a 30 bp overhang homologous to the adjacent *ENO2* promoter
	719	AAGGTAGACCAATGTAGCGCTCTTACTTTATTATTCAGA AACATCTTCATCTTGACCTG	Primer for Gibson Assembly - amplification of *An*DCT-02 from pGBS416DCT-2 with a 30 bp overhang homologous to the adjacent *DIT1* terminator
	720	CCAGGTCAAGATGAAGATGTTTCTGAATAATAAAGTAA GAGCGCTACATTGGTCT	Primer Gibson Assembly- amplification of *DIT1* terminator from *S. cerevisiae* Sc288c DNA with a 30 bp overhang homologous to the adjacent *AnDCT*-02 open reading frame
	309	GAAACAGCTATGACCATGATTACGAATTCGAGCTCGGT ACCCGGGTTACTCCGCAACGCTTTTCTGAACG	Primer Gibson Assembly - for amplification of *DIT1* terminator from *S. cerevisiae* Sc288c DNA with a 40 bp overhang homologous to the adjacent BamHI-linearized-pUC18 vector backbone
**Genomic integrations of expression cassettes employing the CRISPR-Cas9 system**	690	CTGCACATAATTGAAATAAGGATGTAGTTCAACTTCTAT GAATGCTCGGCGATACGATATGGATCCGAAGTACCTTC AAAGAATG	Primer for amplification of *MDH3* expression cassette with an overhang homologous to a sequence upstream of the YGLCτ3 coding sequence in chromosome VII
	770	AAAAAGGAGTAGAAACATTTTGAAGCTATGGATGGTAA TGATCCGAACTTGG	Primer for amplification of *MDH3* expression cassette with an overhang homologous to the adjacent *TEF1* promoter of the *RoFUMR* expression cassette
	771	AAGGTTCCCCAAGTTCGGATCATTACCATCCATAGCTTC AAAATGTTTCTACTC	Primer for amplification of *RoFUMR* expression cassette with an overhang homologous to the adjacent *IDP1* terminator of the *MDH3* expression cassette
	590	GAAATGGCAGTATTGATAATGATAAACTCGAGGAAAAA CGGGAAGAAAAGG	Primer for amplification of *RoFUMR* expression cassette with an overhang homologous to the adjacent *TDH3* promoter of the *TbFRDg-R* expression cassette
	591	TCTTTCCTTTTCTTCCCGTTTTTCCTCGAGTTTATCATTAT CAATACTGC	Primer for amplification of *TbFRDg-R* expression cassette with an overhang homologous to the adjacent *RPL15A* terminator of the *RoFUMR* expression cassette
	772	TTCCGTCTCTGGCTGAAGGCTCATTTCCATGATGGGGTC ACAATTATTATCGCACGCAAATTAAAGCCTTCGAGC	Primer for amplification of *TbFRDg-R* expression cassette with an overhang homologous to a sequence downstream of the YGLCτ3 coding sequence in chromosome VII

	871	TATGGAAGTATCAAAGGGGACGTTCTTCACCTCCTTGG AAGTGTCGACGCTGCGGGTATAG	Primer for amplification of *An*DCT-02 expression cassette with an overhang homologous to a sequence upstream of the YPRCτ3 coding sequence in chromosome XVI
	872	TTACAATCTAGTCGCAAAAACAAGTACAGTGCTGACGT CCCATCTTACTCCGCAACGCTTTTCTGAACG	Primer for amplification of *An*DCT-02 expression cassette with an overhang homologous to a sequence downstream of the YPRCτ3 coding sequence in chromosome XVI
	991	TATGGAAGTATCAAAGGGGACGTTCTTCACCTCCTTGG AAgatccGAAGTACCTTCAAAGAATG	Primer for amplification of *MDH3* expression cassette with an overhang homologous to a sequence upstream of the YPRCτ3 coding sequence in chromosome XVI
	992	TATAGAGTAAAGAACCCTTTCTATACCCGCAGCGTCGAC ACGCAAATTAAAGCCTTCGAG	Primer for amplification of *TbFRDg-R* expression cassette with an overhang homologous to the adjacent *ENO2* promoter of the *An*DCT-02 expression cassette
	993	GAAGGTTTTGGGACGCTCGAAGGCTTTAATTTGCGTGT CGACGCTGCGGGTATAG	Primer for amplification of *An*DCT-02 expression cassette with an overhang homologous to the adjacent terminator *CYC1* of the *TbFRDg-R* expression cassette
**Deletion of *ICL1* gene**	1189	AACAATTGAGAGAAAACTCTTAGCATAACATAACAAAA AGTCAACGAAAACCAGCTGAAGCTTCGTACGC	For amplification of phleomycin deletion cassette from pUG66 with a sequence complementary to the flanking regions of the genomic integration site at their 5' end and a sequence complementary to the loxP sites on pUG66 at their 3' end.
	1190	ATATACTTGTCAGGAAATGCCGGCAGTTCTAATGGTTA ATCCTTGTCCGCATAGGCCACTAGTGGATCTG	

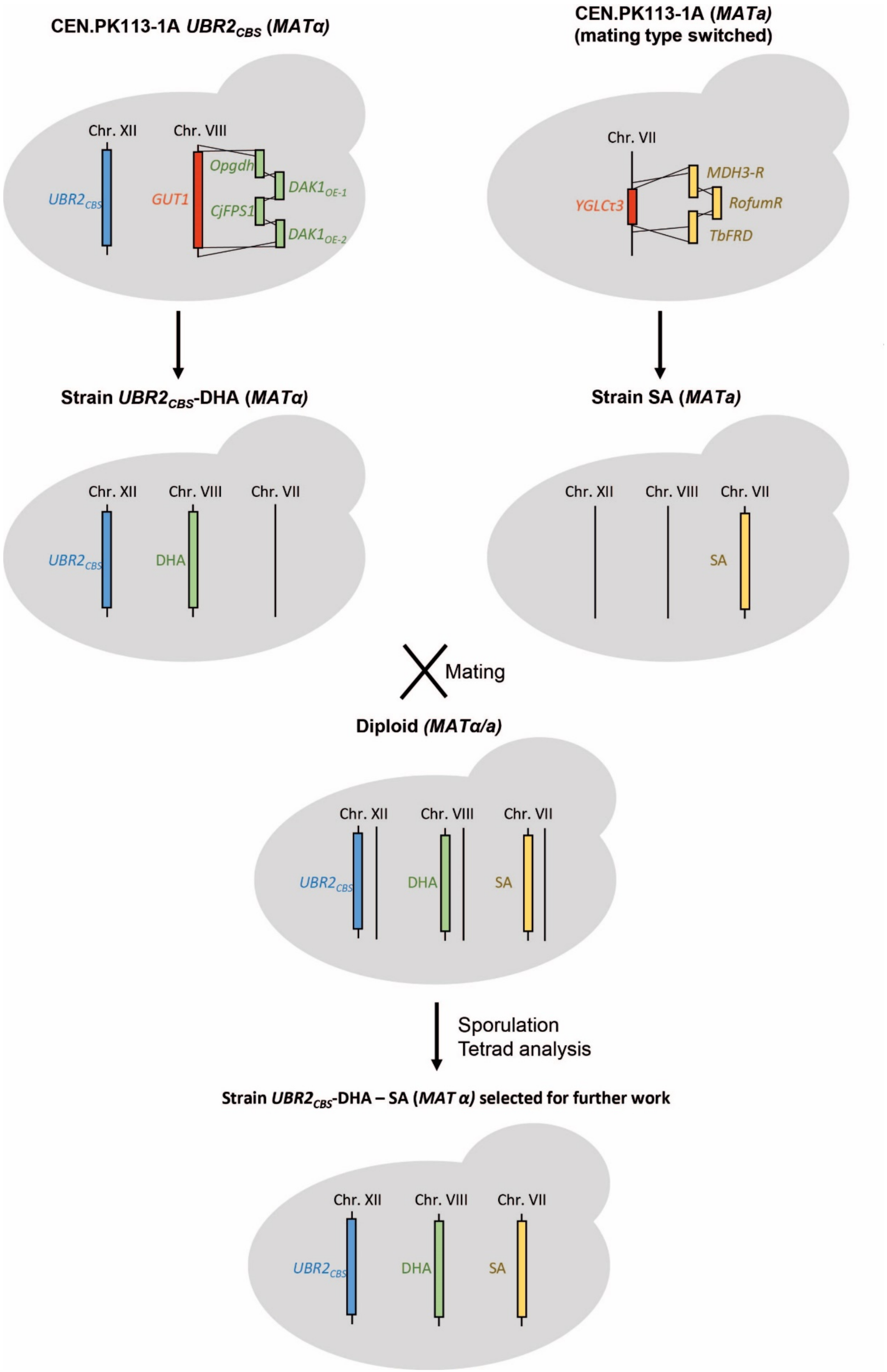

Fig. S1: Mating strategy used for construction of strain *UBR2*$_{CBS}$-DHA-SA.

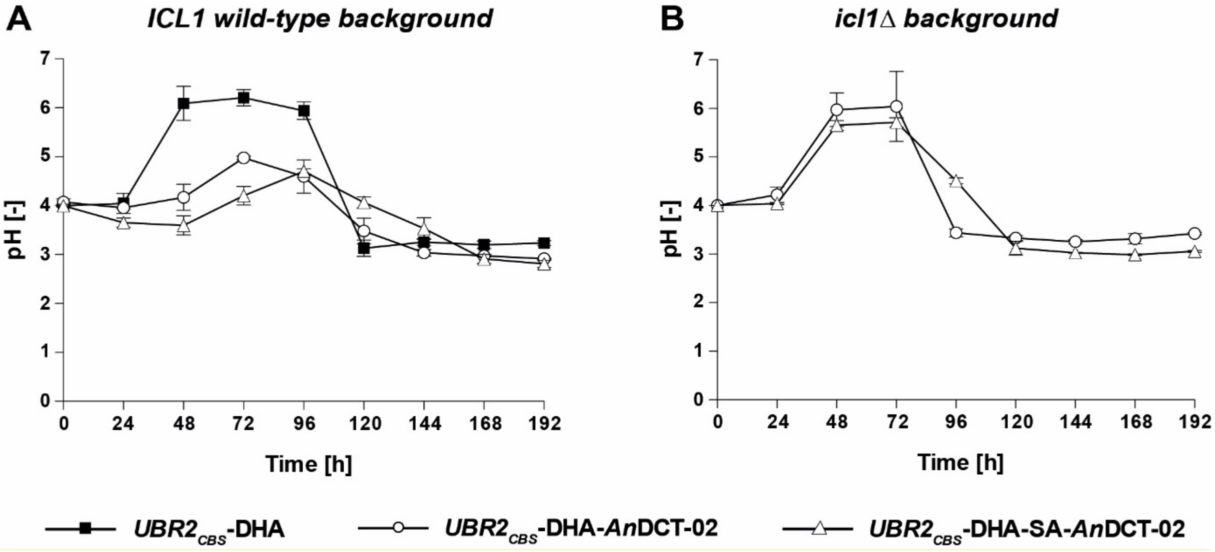

Fig. S2: pH profile during shake flasks (batch) cultivation in *S. cerevisiae* strains *UBR2$_{CBS}$*-DHA, *UBR2$_{CBS}$*-DHA-*An*DCT-02 and *UBR2$_{CBS}$*-DHA-SA-*An*DCT-02 (A) and in strains *UBR2$_{CBS}$*-DHA-*An*DCT-02 *icl1*Δ and *UBR2$_{CBS}$*-DHA-SA-*An*DCT-02 *icl1*Δ (B). Cultivations were performed in 500 mL shake flasks cultures containing 100 mL of synthetic medium containing 75.6 g/L glycerol as the sole carbon source. Mean values and standard deviations were determined from at least three biological replicates.

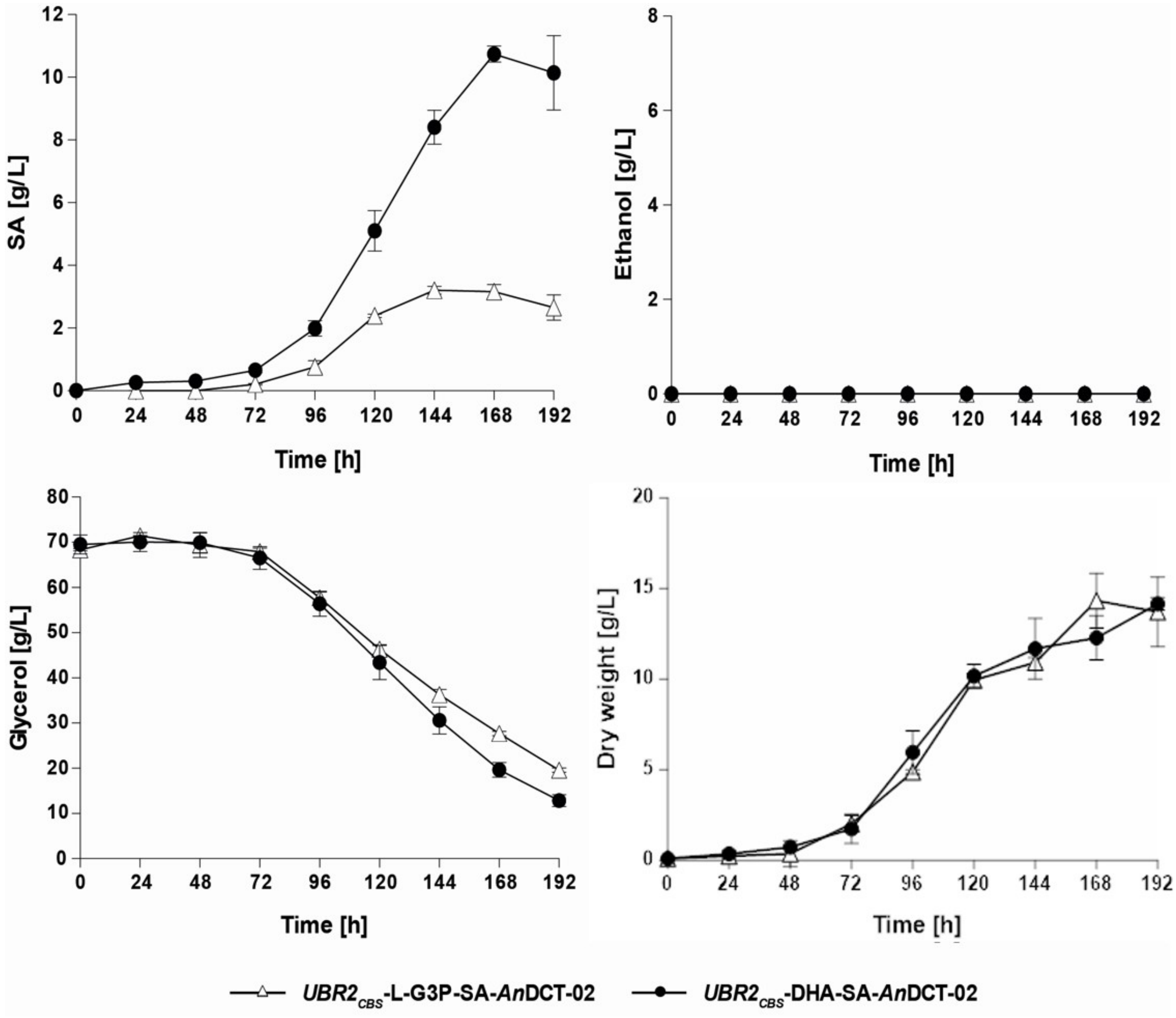

Fig. S3: Comparison of the L-G3P pathway (strain *UBR2*$_{CBS}$-L-G3P-SA-*An*DCT-02) and the DHA pathway (strain *UBR2*$_{CBS}$-DHA-SA-*An*DCT-02) on the fermentation performance and product formation in *S. cerevisiae* in which the reductive SA production pathway and the *An*DCT-02 transporter was established. Cultivations were performed in 500 mL shake flasks cultures containing 100 mL of synthetic medium containing 75.6 g/L glycerol as the sole carbon source and culture supernatants were analysed by HPLC for production of SA, ethanol, and the consumption of glycerol. Growth was recorded by optical density measurements at 600 nm. The culture biomass was determined by correlating OD_{600} measurements to dry weight as described in Material and Methods. Mean values and standard deviations were determined from at least three biological replicates.

References

Gueldener, U., Heinisch, J., Koehler, G. J., Voss, D., Hegemann, J. H., 2002. A second set of loxP marker cassettes for Cre-mediated multiple gene knockouts in budding yeast. Nucleic Acids Research. 30.

Islam, Z.-u., Klein, M., Aßkamp, M. R., Ødum, A. S. R., Nevoigt, E., 2017. A modular metabolic engineering approach for the production of 1,2-propanediol from glycerol by *Saccharomyces cerevisiae*. Metabolic Engineering. 44**,** 223-235.

Klein, M., Carrillo, M., Xiberras, J., Islam, Z., Swinnen, S., Nevoigt, E., 2016. Towards the exploitation of glycerol's high reducing power in *Saccharomyces cerevisiae*-based bioprocesses. Metabolic Engineering. 38.

Yanisch-Perron, C., Vieira, J., Messing, J., 1985. Improved M13 phage cloning vectors and host strains: nucleotide sequences of the M13mp18 and pUC19 vectors. Gene. 33**,** 103-19.
